# Diclofenac and triamcinolone acetonide impair tenocytic differentiation and promote adipocytic differentiation of mesenchymal stem cells

**DOI:** 10.1186/1749-799X-8-30

**Published:** 2013-09-02

**Authors:** Maritha Fredriksson, Yan Li, Anders Stålman, Lars-Arne Haldosén, Li Felländer-Tsai

**Affiliations:** 1 Department of Clinical Intervention, Technology and Science, Karolinska Institutet, Stockholm 141 86, Sweden; 2 Department of Orthopaedics, Karolinska University Hospital Huddinge, Solna 171 76, Sweden; 3 Department of Biosciences and Nutrition, NOVUM, Karolinska Institutet, Stockholm 141 86, Sweden

**Keywords:** Tendinopathy, Triamcinolone acetonide, Diclofenac, Mesenchymal stem cells

## Abstract

**Background:**

Tendinopathies are often empirically treated with oral/topical nonsteroidal anti-inflammatory medications and corticosteroid injections despite their unclear effects on tendon regeneration. Recent studies indicate that tendon progenitors exhibit stem cell-like properties, i.e., differentiation to osteoblasts, adipocytes, and chondrocytes, in addition to tenocytes. Our present study aims at understanding the effects of triamcinolone acetonide and diclofenac on tenocytic differentiation of mesenchymal stem cells.

**Methods:**

The murine fibroblast C3H10T1/2 cell line was induced to tenocytic differentiation by growth differentiation factor-7. Cell proliferation and differentiation with the exposure of different concentrations of triamcinolone acetonide and diclofenac were measured by WST-1 assay and real-time polymerase chain reaction analysis, respectively.

**Results:**

Cell proliferation was decreased in a concentration-dependent manner when exposed to triamcinolone acetonide and diclofenac. In addition to tenocytic differentiation, adipocyte formation was observed, both at gene expression and microscopic level, when the cells were exposed to triamcinolone acetonide or high concentrations of diclofenac.

**Conclusions:**

Our results indicate that triamcinolone acetonide and diclofenac might alter mesenchymal stem cell differentiation in a nonfavorable way regarding tendon regeneration; therefore, these medications should be used with more caution clinically.

## Introduction

Tendons are collagenous connective tissues, which connect bones to muscles and are essential for joint movement. The human tendon can withstand considerable forces during locomotion based on a dense fibrillar network of collagen fibers [1,2]; however, this hypocellular and hypovascular structure also makes the tendon prone to overuse injuries, as what often occurs with repetitive loading in sports and leisure activities [3]. This process is defined as tendinopathy, which is a common orthopedic condition characterized by pain during activity, localized tenderness upon palpation, swelling of the tendon, and impaired performance [4]. Tendinopathies around the larger joints are disabling [5]. The treatments of these conditions are often empirically initiated with oral/topical nonsteroidal anti-inflammatory medications (NSAIDs) and/or corticosteroid injections [6]. The aim is to reduce symptoms and promote mobilization. However, the biological/pharmacological basis of these methods, especially their effects on tendon regeneration, is largely uncertain [7-9].

The repair activity following tendon injury is associated with fibroblast-like cells from the epitenon and endotenon, termed as tenoblasts. They are immature, proliferative cells and are considered as precursors of terminally differentiated tenocytes, which are responsible for matrix synthesis [10]. Recent studies indicate that these ‘niched’ fibroblasts exhibit stem cell-like properties [11], i.e., ability to differentiate into cells of a variety of mesenchymal tissues, such as the bone, fat, and cartilage, in addition to the tendon [12,13]. Interestingly, tendinopathy is histologically characterized with a marked reduction in the amount of healthy tenocytes and the accumulation of ‘non-tendon cells’, including myofibroblasts, adipocytes, chondrocytes, and osteoblasts [14]. Therefore, tendinopathy might result in impairment of tenocytic differentiation of the stem cells which are directed towards other mesenchymal lineages under pathological condition.

Our present *in vitro* study aimed to evaluate the influence of NSAIDs and corticosteroid on tenocytic differentiation of mesenchymal stem cells (MSC). We used the well-established murine mesenchymal cell model C3H10T1/2 where cells were induced to tenocytic differentiation through the treatment with growth differentiation factor-7 (GDF-7), which has been reported to induce tenogenic maturation in tendon-derived cells [15-18]. The effects of diclofenac (DF) and triamcinolone acetonide (TA) on cell proliferation and lineage differentiation were evaluated.

## Methods

### Cell culture

Murine mesenchymal stem cells, C3H10TY1/2 (LGC Standards) were routinely cultured in Dulbecco's BME medium (Invitrogen, Life Technologies, Carlsbad, CA, USA) supplemented with 10% heat-inactivated fetal bovine serum (Saveen Werner, Limhamn, Sweden), 1% gentamicin (Invitrogen) and 1% l-glutamate (Invitrogen) in an incubator with 95% O_2_ and 5% CO_2_.

### Cell proliferation assay

In 96-well plates, 700 cells per well were seeded and allowed to adhere overnight. The next day (day 0), cells were exposed to DF (0.1–100 μM; Sigma) or TA (1 nM–1 μM (Sigma-Aldrich Corporation, St. Louis, MO, USA). The medium was changed every third day. Cell growth was analyzed on days 0, 1, 3, and 5 with WST-1 kit (Roche, Besel, Switzerland), where a tetrazolium salt is cleaved to formazan by cellular enzymes, i.e., mitochondrial succinate dehydrogenase. Increase of enzyme activity leads to increased formazan dye formation, which correlates to the number of metabolically active cells in culture. The formed dye was quantified with SpectraMax 250 microplate reader (Molecular Devices, LLC, Sunnyvale, CA, USA) against a background control.

### Differentiation and treatment groups

Cells plated in six-well plates were differentiated towards tenocytic lineage using GDF-7 (R&D Systems, Minneapolis, MN, USA) at a final concentration of 50 ng/ml. Ascorbic acid was also added to a final concentration of 50 μg/ml to support proper collagen synthesis. From the start of differentiation and up to 9 days of treatment, differentiating cells were exposed to DF (0.1–100 μM) or TA (1 nM–1 μM) and then analyzed for expression of tenocyte, osteoblast, and adipocyte markers as described below.

### Quantitative real-time polymerase chain reaction assay

Cells were grown in six-well culture plastic plates for 0–9 days and then lysed using Trizol reagent (Invitrogen) and RNA, prepared as described by manufacturer. Isolated RNA (1 μg) was subjected to DNase treatment (Invitrogen) and thereafter reverse-transcribed with Super Script II reverse transcriptase to cDNA using random primers (Invitrogen). cDNA was subjected to real-time polymerase chain reaction (RT-PCR) with SYBR Green PCR Master Mix (Applied Biosystems, Foster City, CA, USA) in an ABI PRISM 7500 apparatus (Applied Biosystems). Reaction conditions were 1 cycle at 95°C for 20 s followed by 40 cycles at 90°C for 3 s and at 60°C for 30 s. Primer sequences are described in Table 1.

**Table 1:** Sequences of used oligonucleotide primers

**Primer sequence**		
**Targets**	**Forward primer**	**Reverse primer**
Collagen 1α1	gagcggagagtactggatcg	gcttcttttccttggggttc
Tenomodulin	tgtactggatcaatcccactct	gctcattctggtcaactcccct
aP2	catggccaagcccaacat	cgcccagtttgaaggaaatc
Osteocalcin	gccatcaccctgtctcctaaa	gctgtggagaagacacagca
Runx2	gccgggaatgatgagaacta	ggtgaaactcttgcctcgtc

### Statistical methods and data management

Multiple comparisons of continuous data were performed by analysis of variance (ANOVA). In the case of a statistically significant result in the ANOVA, statistical comparisons in order to test differences between groups, *post hoc* comparisons, were made using Dunnett's *post hoc* test when comparing a control group with the other groups, and the *post hoc* procedure proposed by Bonferroni was used to control for multiplicity when comparing arbitrary groups. All analyses were carried out using the SPSS system.

## Results

### Effect of TA and DF on proliferation of MSC

**Figure 1:**
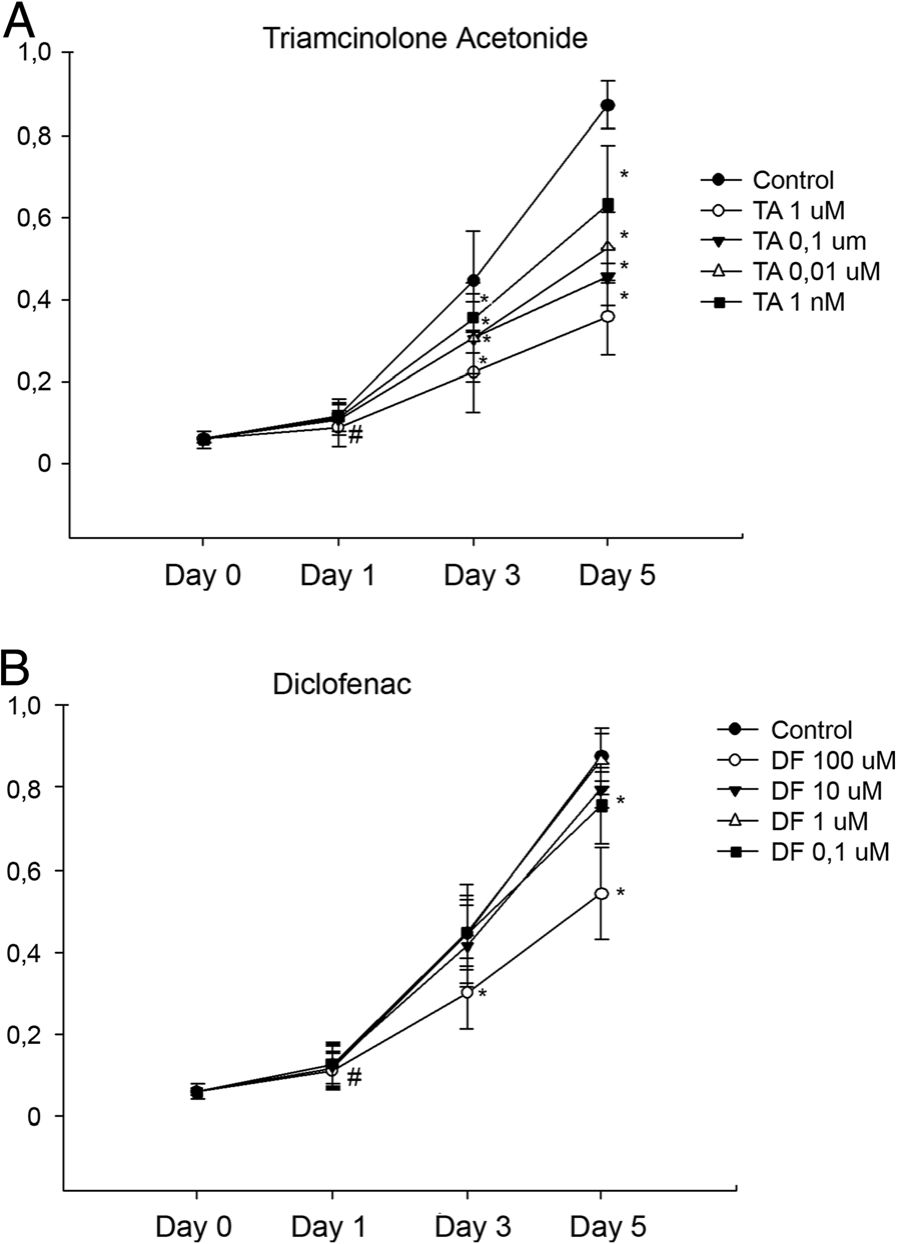
**Effect of TA and DF on proliferation of MSC.** MSCs were treated with TA **(A)** and DF **(B)** for 5 days. Proliferation was measured on days 0, 1, 3, and 5 using proliferation kit WST-1. Each data point represents the mean ± SD of the three samples (*P < 0.01, ^#^*P* < 0.05).

Exposure of MSC to TA had a negative effect on proliferation, as measured with WST-1 cell proliferation assay (Figure 1). At day 3 of exposure, a negative effect on proliferation was noticed with the tested concentrations of TA. At day 5, all tested concentrations clearly influenced proliferation in a concentration-dependent manner. The NSAID, diclofenac, also decreased cell proliferation on days 3 and 5 but was more significant with the highest concentration tested, at 100 μM (^*#*^*P* < 0.05, *P < 0.01).

### Effect of GDF-7 on differentiation of MSC

**Figure 2:**
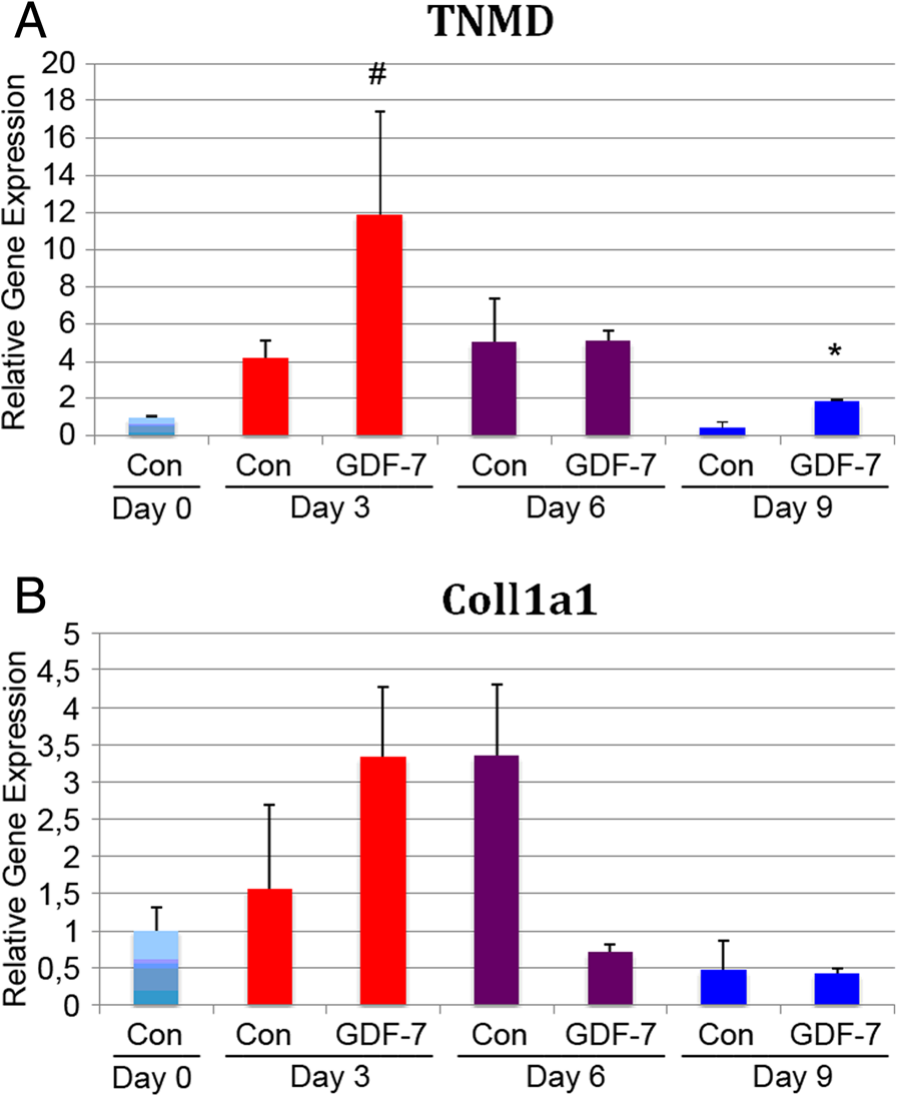
**Effect of GDF-7 on differentiation of MSC to tenocytes.** Cells were grown in the medium with or without GDF-7 (50 ng/ml). Gene expression of tenocyte markers *TNMD***(A)** and *CollIα1***(B)** was analyzed on days 0, 3, 6, and 9 using qPCR. Each data point represents the mean ± SD of the three samples (*P < 0.01, ^#^*P* < 0.05).

The peptide hormone GDF-7 was used to differentiate MSC towards the tenocytic lineage. In Figure 2A,B, it can be seen that GDF-7 increased the expression of the tenocyte markers, tenomodulin (TNMD) and collagen Iα1 (CollIα1) with more apparent effects at early time points (^#^*P* < 0.05, *P < 0.01).

### Effect on tenocytic differentiation of MSC after exposure to TA and DF

**Figure 3:**
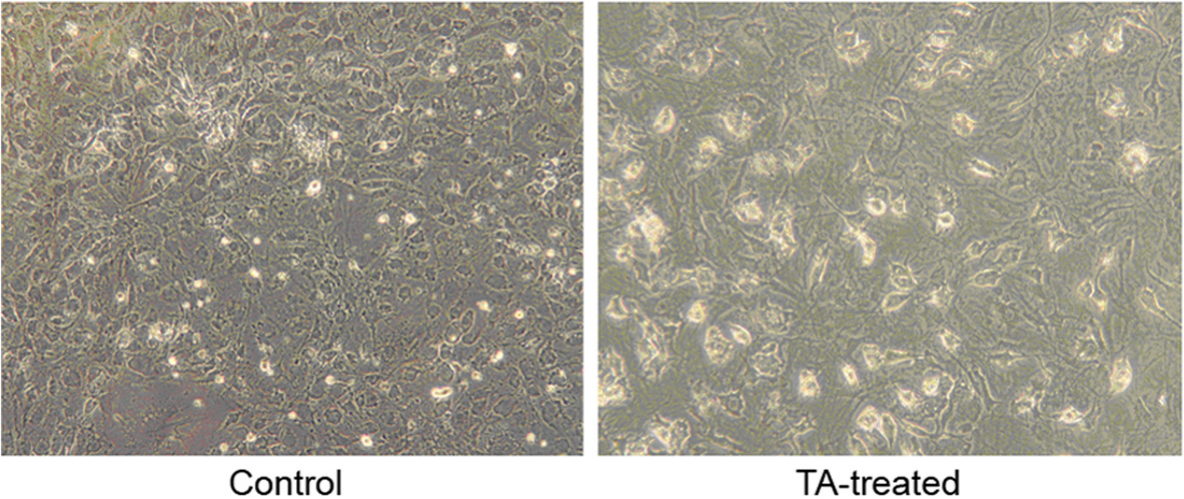
**Photomicrograph of untreated and 100 nM TA-treated MSC after 9 days of culture.** Magnification × 10.

**Figure 4:**
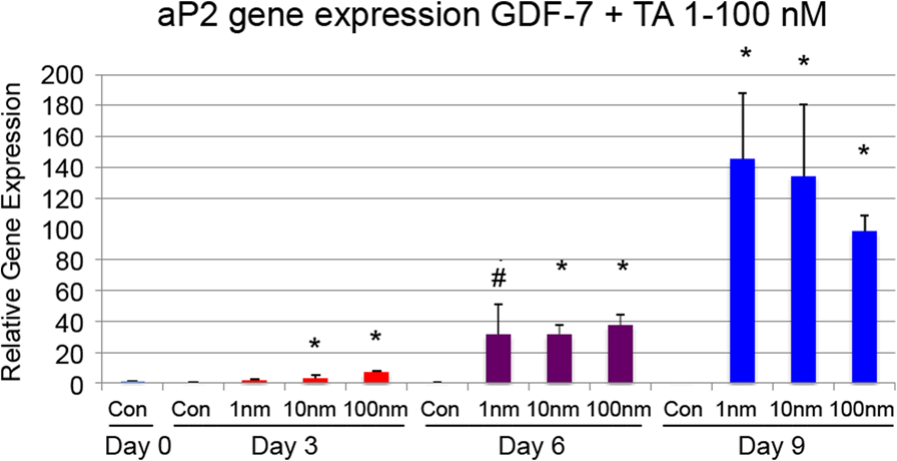
**aP2 gene expression analysis after exposure to TA.** Cells were treated with GDF-7 (50 ng/ml) combined with or without 1, 10, or 100 nM TA. aP2 gene expression was measured on days 0, 3, 6, and 9 using qPCR. Each data point represents the mean ± SD of the three samples (*P < 0.01, ^#^*P* < 0.05).

In preliminary experiments, we found that simultaneous exposure of MSC to GDF-7 and DF or TA significantly increased the appearance of adipocytes in culture dishes. With DF, this was only seen after exposure to the highest concentration tested, at 100 μM (data not shown). Regarding TA, the appearance of adipocytes was seen with all concentrations tested but was most clearly seen with the highest concentration, at 100 nM (compare Figure 3A,B). However, using the more sensitive qPCR technique, it is clearly seen that TA, even at the lowest concentration and at all time points, had a dramatic effect on aP2 gene expression (Figure 4; ^#^*P* < 0.05, *P < 0.01).

## Discussion

NSAIDs and periodic local corticosteroid injections still represent the mainstays for the treatment of tendon-related syndromes, although the majority of these conditions are primarily degenerative rather than inflammatory [19]. Our results showed that, when exposed to triamcinolone acetonide and diclofenac, the differentiation of mesenchymal stem cells to tenocytic lineage was impaired and the differentiation was drawn towards adipocytic lineage.

We used GDF-7 (also named bone morphogenetic protein (BMP)-12) to induce tenocytic differentiation of the fibroblast-like C3H10T1/2 cells. BMPs belong to the transforming growth factor beta superfamily, which is a group of growth factors known to induce growth and differentiation of various cell types [20]. Unlike the major BMP family subtypes, such as BMP-2, BMP-4, BMP-6, and BMP-7, which are essential for bone and cartilage development [21], BMP-12 belongs to a subtype that lacks the propensity to induce bone formation. Instead, molecules of this class, including BMP-12, BMP-13, and GDF-5, were found to induce the formation of tendon-like structures [22]. Consistent with previous reports [15-18], we found that the expression of two tendon-related markers, TNMD and CollIα1, significantly increased with exposure to GDF-7. However, the differentiated cells presented heterogeneous morphological features, and lipid droplets could be clearly seen in part of the cell population. This was further confirmed on the analysis of the expression of the adipocyte marker gene, aP2, which codes for fatty acid binding protein [23]. We consider this finding of particular importance because fatty infiltration in a musculotendinous junction characterizes the histopathological changes following tendon injuries, such as in massive rotator cuff tears (RCTs) [24]. Therefore, our results support the current opinion that tendon stem cells may provide a rich source of adipocytes in fatty degeneration [25]. We consider that adding GDF-7 in C3H10T1/2 cultures might create a concise *in vitro* model to study the biological process of this complex orthopedic phenomenon.

Injectable corticosteroids have traditionally been used to manage a variety of musculoskeletal disorders, although the biological basis and systematic evidence for their benefits are largely lacking. Their side effects are also well documented, such as infection, tendon ruptures, and skin and subcutaneous fat atrophy [9]. In addition, experimental studies suggest that corticosteroids not only damage the ultrastructure of collagen molecules [26] but also inhibit the regenerative properties of tendon stem cells [27]. Surprisingly, this evidence has not prevented the prevalence of injectable corticosteroids in routine management of tendon disorders. For example, a recent survey indicates that 96% of practitioners believe that subacromial corticosteroid injection is efficacious in managing rotator cuff tendinopathy [28]. We studied here the effects of TA, a potent long-acting synthetic corticosteroid, on the GDF-7-C3H10T1/2 cell model. We found that, besides the known inhibitory effects on cell proliferation and tenocytic differentiation, TA dramatically promoted adipogenesis. This side effect deserves attention because fatty degeneration is regarded as the key factor to complicate surgical repair of RCTs (with failure rates reported as high as 94% of patients) [29], and no effective treatment is currently available to reverse its progression [24]. Our results therefore raise the question: should a medication that makes the tendon injury irreparable be used for symptom management in tendinopathies? To find the answer, further research using *in vitro* and animal models are needed.

NSAIDs are widely prescribed to reduce pain and inflammation following soft tissue injuries. They inhibit tissue inflammation by repressing cyclooxygenase activity, thereby leading to a reduction in pro-inflammatory prostaglandin synthesis. Despite the possible gastrointestinal and cardiovascular side effects, some NSAIDs are sold as over-the-counter medications and commonly taken by athletes for traumatic or overload injuries [30]. DF is one of these commonly used NSAIDs. It has a fast analgesic onset and a long duration of action. Compared with the other nonspecific NSAIDs, DF is well tolerated and rarely shows serious side effects [31]. In the present study, we found that the highest concentration (100 μM) of DF significantly impaired cell growth and induced adipogenesis, but such effects could not be seen at lower concentrations (0.1–10 μM). This finding is consistent with the previous *in vitro* study of Riley et al. which showed that 2 μg/ml (6.7 μM) of DF had no negative influence on tendon cell proliferation and glycosaminoglycan synthesis [32]. However, due to the limited number of tendon samples, only a single concentration of each drug was tested in their study. Since the serum and soft tissue concentrations of DF after oral intake or topical application are reported to be seldom higher than 10 μM [33,34], further studies are needed to identify whether tendon detrimental effects could really happen following oral or topical application of DF. Up to know, the only *in vivo* study comes from Marsolais et al. who found that the mechanical properties of injured rat Achilles tendons were identical in placebo and diclofenac-treated groups [35]. However, intramuscular DF injection is also popular for the management of postoperative pain [36], and local DF injection has been reported to manage hand tenosynovitis [37]. In both conditions, the para- or intratendinous DF concentration might be higher than 100 μM. Therefore, side effects on tendons should be taken into account when evaluating the safety of these procedures.

Taken together, we established an *in vitro* model to study tenocyte differentiation from their multipotent progenitors. Our results showed that pharmaceuticals such as DF and TA which are commonly used in the symptomatic treatment of musculoskeletal disorders may have profound negative effects on tendon regeneration on a progenitor cell level and therefore should be used with caution.

## Competing interests

The authors declare that they have no competing interests.

## Authors’ contributions

MF conducted the experiments and analyzed the data. YL and MF drafted the manuscript. LAH and LFT designed the experiments and modified the manuscript. AS provided valuable comments for manuscript drafting. All authors read and approved the final manuscript.
